# Withholding or withdrawal of treatment under French rules: a study performed in 43 intensive care units

**DOI:** 10.1186/s13613-015-0056-x

**Published:** 2015-06-19

**Authors:** Olivier Lesieur, Maxime Leloup, Frédéric Gonzalez, Marie-France Mamzer, EPILAT study group

**Affiliations:** Réanimation, CH Saint-Louis, 17019 La Rochelle, France; EA 4569, Université Paris Descartes, 75006 Paris, France; Réanimation Médico-Chirurgicale, CHU Avicenne, 93000 Bobigny, France

**Keywords:** Withholding treatment, Life support care, Medical futility, Prognosis, Brain injury, Advance directives

## Abstract

**Background:**

In France, decisions to limit treatment fall under the Leonetti law adopted in 2005. Leading figures from the French world of politics, science, and justice recently claimed for amendments to the law, considering it incomplete. This study, conducted before any legislative change, aimed to investigate the procedural aspects of withholding/withdrawing treatment in French ICUs and their adequacy with the existing law.

**Methods:**

The characteristics of patients qualified for a withholding/withdrawal procedure were prospectively collected in 43 French ICUs. The study period (60 or 90 days under normal operating conditions) took place in the first half of 2013.

**Results:**

During the study period, 777 (14 %) of 5589 admitted patients and 584 (52 %) of 1132 patients dying in the ICU had their treatment withheld or withdrawn. Whereas 344 patients had treatment(s) withheld (i.e., not started or not increased if already engaged), 433 had one or more treatment(s) withdrawn. Withdrawal of treatment was applied in 156 of 223 (70 %) brain-injured patients, compared to 277 of 554 (50 %) patients with other reasons for admission (*p* < 0.01). At the time of the decision-making, the patient’s wishes were known in 181 (23 %) of the 777 cases in one or more different way(s): 73 (9.4 %) from the patient, 10 (1.3 %) by advance directives, 10 (1.3 %) through a designated trusted person, and 108 (13.9 %) reported by the family or close relatives. An external consultant was involved in less than half of all decisions (356 of 777, 46 %). Of the 777 patients qualified for a withholding/withdrawal procedure, 133 (17 %) were discharged alive from the hospital (126 after withholding, 7 after withdrawal).

**Conclusions:**

More than half of deaths in the study population occurred after a decision to withhold or withdraw treatment. Among patients under withholding/withdrawal procedures, brain-injured subjects were more likely to undergo a withdrawal procedure. The prevalence of advance directives and designated trusted persons was low. Because patients’ preferences were unknown in more than three quarters of cases, decisions remained primarily based on medical judgment. Limitations, especially withholding of treatment, did not preclude survival and hospital discharge.

## Background

Several decades ago, most patients who suddenly died in the hospital underwent cardiopulmonary resuscitation before death. From the 1950s onward, the development of artificial life-sustaining and/or organ-substituting techniques delivered in intensive care units (ICUs) has shifted the definition of death from a sudden and unexpected event to a partially controlled process. Far from searching to avoid death “at any cost” irrespective of the ensuing living conditions, the primary goal of intensive care is to return the highest number of critically ill patients to a quality of life they would find acceptable [1]. Because interfering in the dying process is not always in the patient’s best interest, life-prolonging therapies may be withheld or withdrawn when they are deemed “futile.”

Substantial variability exists between countries, institutions, and physicians in the decision to withhold or withdraw (WhWd) life-sustaining treatment in critically ill patients [2–10]. At an individual level, available prognostic indexes are not accurate enough to make definite end-of-life decisions without foretelling a destiny that would become self-fulfilling (“self-fulfilling prophecy”) [5, 11, 12]. Moreover, withholding/withdrawal decision-making may be considered by stakeholders (patients, relatives, and caregivers) with different hopes and preferences influenced by age, gender, religion, culture, education, training, and geography [5, 10, 13–20]. Legislation, case mix, availability of ICU resources, and organization of care in the institution/region may also influence physicians’ attitude towards end-of-life care [4, 21]. Furthermore, the lack of a consensus-based model for decision-making may favor variability in withholding/withdrawal decisions, published guidelines mainly focusing on general principles rather than practical details [22, 23].

In France, the decision to withhold or withdraw treatment falls under the law n° 2005–370 of April 22, 2005 related to patients’ rights and to the end-of-life (so-called Leonetti law), which authorizes the withholding or withdrawal of curative therapies when deemed “*useless*, *disproportionate or to have no other effect than solely the artificial preservation of life*” [24]. Continuing such treatment with no hope of benefit or cure would equate to an undue therapeutic obstinacy, especially for patients who are no longer able to express their wishes. In this setting, when current or further life-sustaining treatments appear to be of no overall benefit for a patient, the law stipulates that any WhWd decision should only be made after a formal procedure of collegial deliberation [23, 24]. Obtaining an external opinion from an independent consultant is a compulsory part of the procedure. In addition to medical factors, the Leonetti law specifies that the discussion must integrate the patient’s wishes spontaneously expressed or written in advance directives, the opinion of the trusted person (if appointed), and the family and/or close relatives.

Leading figures from the French world of politics, science, and law recently claimed for amendments to the law reinforcing patients’ rights. The aims of this prospective observational study, conducted before any legislative changes, were to investigate the incidence of withholding/withdrawal decisions in French ICUs and to evaluate how these procedures were implemented with regard to the legislation in force.

## Methods

This report is a secondary analysis of a previous study designed to assess the theoretical eligibility as organ donors of patients deceased after end-of-life decisions [25]. The study was performed in 43 French ICUs (15 units in university-affiliated centers, 28 in general hospitals). The institutional review board (CPP Paris Ile de France II, IRB registration: 00001072) approved the protocol. The study period (60 or 90 consecutive days in normal operating conditions) took place during the first half of 2013. All patients admitted to the ICU who underwent a WhWd procedure according to the terms of the French Leonetti law were enrolled in the survey.

The epidemiological data recorded during the ICU stay included age, gender, medical history, reasons for admission, Simplified Acute Physiology Score (SAPS) II index [26], Knaus [27], McCabe [28], and Charlson [29] scores on admission, relevant clinical and biological characteristics, Sequential Organ Failure Assessment (SOFA) score [30] at the time of the WhWd decision, reasons for the decision, implemented measures, and outcome of the patient (deceased or discharged alive). By convention, a SOFA organ sub-score of 3 or more was considered as an organ failure. WhWd patients discharged from the ICU were followed until discharge from the hospital or death in the ward.

For WhWd patients, we analyzed how the limitations were carried out with regard to the contribution of caregivers in end-of-life decisions (including consultant physicians), patient or surrogate decision-maker involvement, and advance directives. Reasons for limiting treatment (based on items proposed by the French intensive care society [23]), participants in the decision-making process, and type of treatment withheld or withdrawn were registered by the local investigator using a form developed for this purpose.

Limitations differentially included withholding or/and withdrawing therapies like cardiopulmonary resuscitation, endotracheal intubation, ventilatory support, renal replacement therapy, inotrope use, urgent surgery, antimicrobial therapy, blood product transfusion, nutrition, and hydration. A three-level hierarchy for classifying decisions used the more active mode of limitation (“stop” > “do not increase” > “do not start”) if more than one was performed. “Withdrawing” was defined as the decision to stop a treatment already undertaken. “Withholding” was defined as the decision not to start or increase a treatment beyond a critical threshold. Patients were classified as “withheld (Wh) patient” if withholding was the single limitation made and as “withdrawn (Wd) patient” if treatments were both withheld and withdrawn. Within the Wh sub-group, the patients who had one or more “do not increase” order(s) were compared to those exclusively qualified for “do not start” instruction(s).

### Statistical analysis

Results are expressed as mean ± standard deviation (SD) or median and interquartile (IQR) for continuous variables and percentage with 95 % confidence interval (CI) for categorical variables. Simple regression analysis was used to establish the relationship between continuous variables (SAPS II, mortality rate) and the WhWd decision rates among the 43 participating ICUs. Comparisons of patients were based on *t* test or the Mann–Whitney *U* test for continuous variables and on chi-square (χ^2^) test or Fisher’s exact test for categorical variables, as appropriate. A two-tailed *p* value < 0.05 was considered statistically significant. We used univariate and multivariate logistic regression analyses with Wh/Wd as a binary procedural variable to assess associations with categorical variables. All relevant univariate indexes with *p* value less than 0.2 were included in the multivariate logistic regression model (age > 70 years, comorbidities, reasons for admission, organ failures). Descriptive statistics, univariate, and multivariate regressions were performed using Epi Info^TM^ (Centers for Disease Control and Prevention, Atlanta, GA) and the R statistical package (R Core Team, R Foundation for Statistical Computing, Vienna, Austria).

## Results

### Study population

During the study period, 5589 patients (age: 62 ± 17 years; gender ratio M/F: 1.6; SAPS II: 44 ± 22) were admitted to 43 ICUs (616 beds). A total of 4457 patients (80 %) were discharged alive from the ICU. One thousand one hundred thirty-two patients (20 %) died in the ICU. The median (IQR) mortality rate across the 43 participating ICUs was 20.8 (17–25) %. Over half of the deceased patients (584/1132, 52 %) underwent a formalized WhWd procedure before death.

**Fig. 1:**
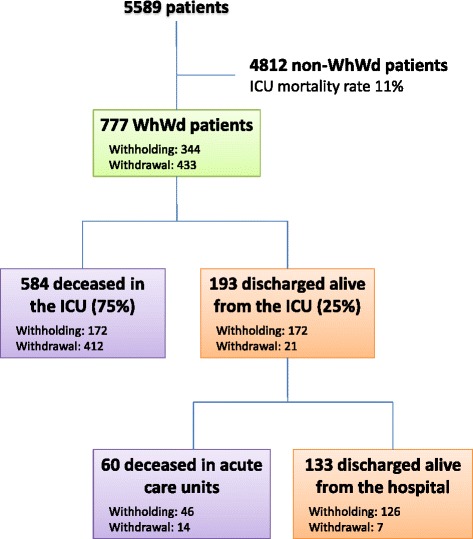
Flow chart. *WhWd* withhold or withdraw treatment, *ICU* intensive care unit

**Fig. 2:**
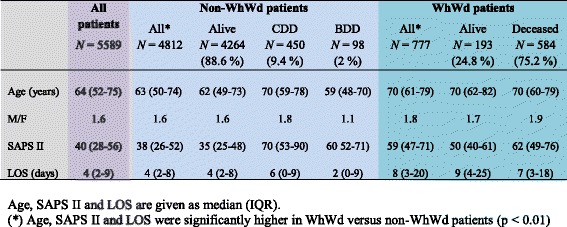
Characteristics of patients admitted over the study period. *WhWd* withhold or withdraw treatment, *Alive* discharged alive from the ICU, *CDD* circulatory determination of death, *BDD* brain determination of death, *deceased* in the ICU, *M*/*F* sex ratio, *SAPS* Simplified Acute Physiology Score, *LOS* length of stay

**Fig. 3:**
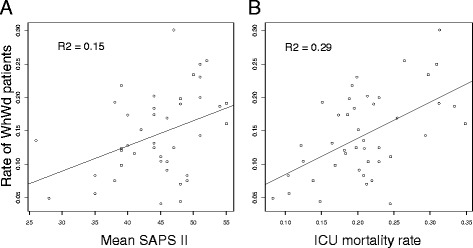
Relationship between SAPS II (**a**), mortality rate (**b**), and WhWd decision rate among the 43 participating ICUs. *SAPS* Simplified Acute Physiology Score, *WhWd* withhold or withdraw treatment, *ICU* intensive care unit

Of the 5589 patients admitted, 777 (14 %; age: 68 ± 14 years; gender ratio M/F: 1.8; SAPS II: 60 ± 20; SOFA: 7 [4–11]) underwent Wh (344 patients, 6 %) and/or Wd (433 patients, 8 %) measures (Figs. 1 and 2). Table 1 shows the study population for each participating ICU with regard to end-of-life decisions and outcome (deceased or discharged alive). The median (IQR), minimum, and maximum proportions of WhWd patients across the 43 ICUs were 13.2 (10–19), 4, and 30 %, respectively. The proportion of WhWd patients across the 43 ICUs correlated with SAPS II (*p* < 0.02, Fig. 3a) and mortality rates (*p* < 0.001, Fig. 3b). Baseline data, reasons for admission, and outcome of the 777 WhWd patients are shown in Tables 2 (all) and 3 (separating Wh and Wd).

**Table 1:** Study population in the 43 participating ICUs

ICU	Hospital	ICU beds, *N*	ICU admissions, *N*	Mean age, years	Mean SAPS II	In-ICU death rate, %	WhWd patients, *N* (% admissions)	Wh/Wd patients *N*/*N*	Discharged alive from the ICU after WhWd, *N* (% WhWd)
1	General	12	48	58	35	10	4 (8)	1/3	1 (25)
2	General	10	48	68	49	21	4 (8)	2/2	2 (50)
3	General	8	49	64	45	24	2 (4)	0/2	1 (50)
4	General	12	63	67	48	22	12 (19)	1/11	0 (0)
5	University	9	71	52	39	17	7 (10)	0/7	0 (0)
6	General	8	78	62	39	19	17 (22)	14/3	6 (35)
7	General	12	83	70	47	31	25 (30)	11/14	5 (20)
8	General	10	86	64	44	19	15 (17)	11/4	4 (27)
9	General	8	87	66	44	20	16 (18)	14/2	4 (25)
10	General	8	89	63	44	21	18 (20)	9/9	5 (28)
11	University	15	96	61	44	23	12 (13)	5/7	2 (17)
12	General	10	96	64	47	20	12 (13)	5/7	4 (33)
13	General	12	99	61	48	21	7 (7)	1/6	1 (14)
14	General	12	102	64	52	26	26 (25)	17/9	12 (46)
15	University	15	106	65	48	19	21 (20)	2/19	1 (5)
16	General	12	109	68	46	20	10 (9)	2/8	1 (10)
17	General	17	111	61	50	30	26 (23)	15/11	10 (38)
18	University	15	112	57	55	35	18 (16)	7/11	4 (22)
19	University	15	118	59	46	25	20 (17)	5/15	3 (15)
20	General	12	119	58	38	15	23 (19)	11/12	8 (35)
21	General	10	121	60	40	17	21 (17)	14/7	3 (14)
22	University	14	123	66	54	33	23 (19)	8/15	4 (17)
23	University	12	125	50	26	21	17 (14)	2/15	0 (0)
24	General	14	126	61	51	29	18 (14)	1/17	0 (0)
25	General	12	129	63	39	21	16 (12)	3/13	2 (13)
26	University	26	132	56	39	18	16 (12)	7/9	8 (50)
27	General	15	132	61	49	22	10 (8)	4/6	1 (10)
28	General	10	135	69	48	15	6 (4)	3/3	2 (33)
29	General	18	135	63	51	23	27 (20)	9/18	9 (33)
30	General	16	141	66	55	31	27 (19)	23/4	7 (26)
31	General	12	152	65	51	31	38 (25)	23/15	14 (37)
32	General	23	154	58	46	21	16 (10)	7/9	7 (44)
33	University	12	156	59	40	12	20 (13)	9/11	6 (30)
34	General	22	156	64	51	20	36 (23)	20/16	16 (44)
35	University	15	159	58	38	14	12 (8)	9/3	2 (17)
36	University	22	171	59	45	25	19 (11)	6/13	0 (0)
37	University	11	181	69	28	8	9 (5)	3/6	4 (44)
38	University	20	182	57	44	16	24 (13)	7/17	8 (33)
39	General	18	183	65	46	23	19 (10)	8/11	2 (11)
40	General	25	224	62	42	20	34 (15)	5/29	2 (6)
41	General	18	240	60	41	18	28 (12)	13/15	7 (25)
42	University	15	266	59	35	11	15 (6)	13/2	6 (40)
43	University	24	296	61	45	13	31 (10)	14/17	9 (29)
	All	616	5589				777 (14)	344/433	193(25)

*SAPS* Simplified Acute Physiology Score, *WhWd* withhold or withdraw treatment, *ICU* intensive care unit

**Table 2:** Baseline data of the 777 WhWd patients

		Number	Percent	95 % CI
Knaus	A	185	24	21.0–27.2
	B	224	29	25.9–32.4
	C	276	35.8	32.4–39.3
	D	87	11.3	9.2–13.8
	MD	5		
Mc Cabe	0	315	40.9	37.4–44.4
	1	299	38.8	35.3–42.3
	2	157	20.4	17.6–23.4
	MD	6		
Risk factors				
Hypertension		385	49.6	46.0–53.2
Tobacco		238	30.7	27.5–34.1
Diabetes		183	23.6	20.7–26.8
Dyslipidemia		175	22.6	19.7–25.7
Alcohol		159	20.5	17.7–23.5
Chronic diseases				
Cardiac		282	36.3	32.9–39.8
Pulmonary		235	30.2	27.1–33.6
Neurological		130	16.7	14.2–19.6
Renal		109	14.0	11.7–16.7
Vascular		101	13.0	10.8–15.6
Hepatic		77	9.9	7.9–12.3
Intestinal		54	6.9	5.3–9.0
Neurological deficit				
Cognition		94	12.1	9.9–14.6
Swallowing		46	5.9	4.4–7.9
Hemiplegia		22	2.8	1.8–4.3
Tetraplegia		11	1.4	0.7–2.6
Malignancies		221	28.4	25.3–31.8
Rare diseases		44	5.7	4.2–7.6
Reason for ICU admission				
Respiratory failure		259	33.3	30.0–36.8
Shock and MOF		215	27.7	24.6–31.0
Post-cardiac arrest coma		150	19.3	16.6–22.3
Stroke		49	6.3	4.7–8.3
Head trauma		24	3.1	2.0–4.6
Other		80	10.3	8.3–12.7

*WhWd* withhold or withdraw treatment, *ICU* intensive care unit, *MOF* multiple organ failure, *CI* confidence interval, *MD* missing data

### WhWd decision-making

Whatever the procedure (Wh or Wd), the median (IQR) time from the ICU admission to the WhWd decision was 4 (1–13) days. At the time of the decision-making, the patient’s wishes were known in 181 cases (23 %), on the basis of one or more different source(s) of information: spontaneously voiced (73 cases, 9.4 %), written in advance directives (10 cases, 1.3 %), expressed by a designated trusted person (10 cases, 1.3 %), and/or reported by the family or close relatives (108 cases, 13.9 %). An external consultant physician was involved in the decision-making process in less than half of the 777 cases (356 patients, 46 %): 142/344 (41.3 %) and 214/433 (49.4 %) cases for Wh and Wd, respectively (*p* < 0.05).

The rationales most often claimed to justify the WhWd decision were the following:

1. No additional information needed for decision-making: 602 patients (77 %)
2. Limited subsequent functional autonomy: 581 patients (75 %)
3. Absence of curative strategy: 559 patients (72 %)
4. Non-responsive to medical therapy: 516 patients (66 %)
5. Advanced or terminal stage of a severe and incurable disease: 474 patients (61 %)
6. Limited subsequent relational quality of life: 442 patients (57 %)
7. Limited functional autonomy before hospital admission: 317 patients (41 %)
8. Very advanced age: 210 patients (27 %)
9. Perception of disproportionate and non-beneficial treatment voiced by patient’s relatives: 172 patients (22 %)
10. Wish to limit treatment voiced by patient: 110 patients (14 %)

Table 4 shows organ failures, treatment engaged, and rationales for WhWd at the time of the decision-making, separating Wh and Wd.

### Implemented measures

**Fig. 4:**
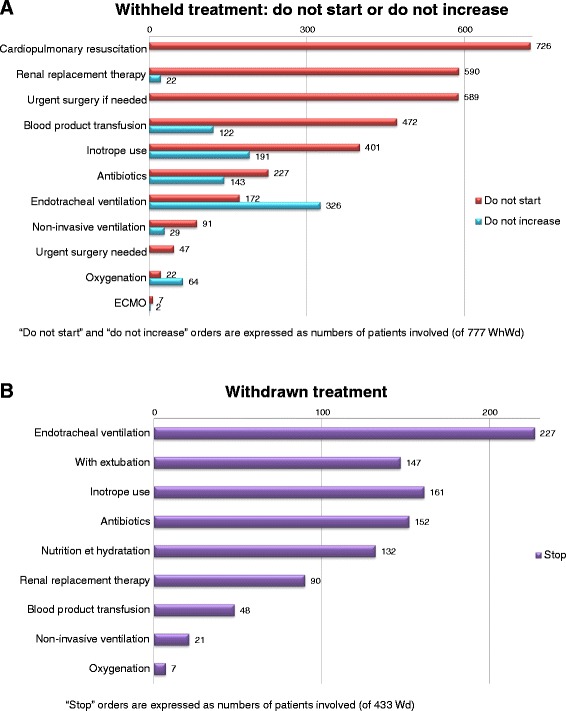
WhWd measures implemented separating “do not start,” “do not increase,” (**a**) and “stop” (**b**) orders. *WhWd* withhold or withdraw treatment, *Wd* withdraw treatment

The WhWd measures implemented are detailed in Fig. 4 separating “do not start,” “do not increase” (Fig. 4a), and “stop” orders (Fig. 4b). After a first WhWd order, 89 patients (11.5 %) received additional measures for limitation of therapy. Endotracheal ventilation was the life-sustaining treatment most often engaged at the time of the decision-making (375 patients) and subsequently withdrawn (227), with (147) or without (80) removal of the endotracheal tube.

Withdrawal of treatment was applied in 156 of 223 (70 %) brain-injured patients, compared to 277 of 554 (50 %) patients with other reasons for admission pooled, who had treatment equally withheld or withdrawn (*p* < 0.01). WhWd patients with chronic respiratory diseases and/or respiratory failure as reason for admission had treatment preferentially withheld than withdrawn (Table 3). For the WhWd patients classified C or D on the Knaus scale, those with cognitive impairment (Table 3), and/or those for whom “limited autonomy before admission” was a rationale to justify WhWd (Table 4), treatment was more likely to be withheld than withdrawn. In a multivariate analysis, neurological (OR 4.5; 95 % CI 3.3–6.2), hematological (2.4; 1.3–4.6), renal (1.8; 1.2–2.6), and circulatory (1.5; 1.1–2.1) failures at the time of the decision were significantly associated with Wd vs Wh.

**Table 3:** Baseline data and outcome of the 777 WhWd patients, separating withholding and withdrawal of treatment

	Withholding,	Withdrawal,	*p*
	*N* = 344	*N* = 433	
Age (years) median (IQR)	71 (62–81)	69 (60–78)	0.03
Male	222 (64.5)	282 (65.1)	0.9
SAPS II median (IQR)	55 (44–70.5)	62 (50–75)	<0.01
Knaus C and D	188 (54.7)	175 (40.4)	<0.01
McCabe = 0	129 (37.5)	186 (43.0)	0.12
Charlson score	5 (3–6)	5 (3–6)	0.5
Chronic diseases:			
Cardiac	135 (39.2)	147 (33.9)	0.13
Pulmonary	128 (37.2)	107 (24.7)	<0.01
Neurological	63 (18.3)	67 (15.5)	0.29
Renal	50 (14.5)	59 (13.6)	0.71
Vascular	43 (12.5)	58 (13.4)	0.71
Hepatic	30 (8.7)	47 (10.9)	0.32
Intestinal	26 (7.6)	28 (6.5)	0.32
Neurological deficit:			
Cognition	51 (14.5)	43 (9.9)	0.02
Swallowing	24 (7.0)	22 (5.1)	0.17
Hemiplegia	9 (2.6)	13 (3.0)	0.46
Tetraplegia	7 (2.0)	4 (0.9)	0.16
Malignancies	90 (26.2)	131 (30.3)	0.20
Rare diseases	17 (4.9)	27 (6.2)	0.27
Reasons for admission:			
Respiratory failure	139 (40.4)	120 (27.7)	<0.01
Shock and MOF	90 (26.2)	125 (28.9)	0.4
Brain injury	67 (19.5)	156 (36.0)	<0.01
Post-cardiac arrest	48 (14.0)	102 (23.6)	<0.01
Stroke	13 (3.8)	36 (8.3)	<0.01
Head trauma	6 (1.7)	18 (4.2)	0.04
Other	48 (14.0)	32 (7.4)	<0.01
LOS (days) in the ICU median (IQR)	9.5 (4–24)	7 (3–17.5)	<0.01
Discharge alive from the ICU	172 (50.0)	21 (4.8)	<0.01
Delay WhWd to the last day in the ICU median (IQR)	3 (1–9)	1 (0–3)	<0.01

Values are represented as number (%), unless stated otherwise.

*WhWd* withhold or withdraw treatment, *SAPS* Simplified Acute Physiology Score, *ICU* intensive care unit, *MOF* multiple organ failure, *LOS* length of stay

**Table 4:** Organ failures, treatment already engaged, and rationales for WhWd at the time of the decision-making, separating withholding and withdrawal of treatment

	Withholding,	Withdrawal,	*p*
	*N* = 344	*N* = 433	
Total SOFA score median (IQR)	5 (3–9)	9 (5–13)	<0.01
Total SOFA score ≥ 8	122 (35.5)	260 (60.0)	<0.01
Total SOFA score ≥ 5	198 (57.6)	367 (84.8)	<0.01
Total SOFA score ≥ 3	282 (82.0)	418 (96.5)	<0.01
Respiratory failure	119 (34.6)	182 (42.0)	0.03
Neurologic failure	104 (30.2)	281 (64.9)	<0.01
Circulatory failure	103 (29.9)	192 (44.3)	<0.01
Hepatic failure	10 (2.9)	37 (8.5)	<0.01
Hematologic failure	14 (4.1)	54 (12.5)	<0.01
Renal failure	56 (16.3)	118 (27.3)	<0.01
Treatments already engaged:			
Endotracheal ventilation	213 (61.9)	375 (86.6)	<0.01
Non-invasive ventilation	50 (27.9)	35 (17.3)	<0.01
Inotrope use	107 (31.1)	206 (47.6)	<0.01
Renal replacement therapy	24 (7.2)	100 (23.6)	<0.01
Antibiotics	158 (45.9)	256 (59.1)	<0.01
Blood product transfusion	46 (14.9)	77 (18.6)	0.11
Surgery needed	22 (6.4)	47 (10.9)	0.02
Rationales to justify WhWd:			
No additional information needed	246 (78.1)	356 (90.8)	<0.01
Limited subsequent autonomy	252 (80.3)	329 (81.4)	0.34
Absence of curative therapy	199 (61.4)	360 (86.5)	<0.01
Non-responsive to treatment	180 (56.6)	336 (83.6)	<0.01
End-stage incurable severe disease	185 (58.4)	289 (73.5)	<0.01
Limited subsequent relational QOL	177 (57.7)	265 (68.3)	<0.01
Limited autonomy before admission	184 (55.9)	133 (33.8)	<0.01
Very advanced age	101 (31.9)	109 (27.9)	0.14
Excessive treatment felt by relatives	59 (18.9)	113 (28.2)	<0.01
Patient’s wish to limit treatment	59 (19.1)	51 (13.1)	0.02
Awareness of patient’s preferences	86 (25.0)	95 (21.9)	0.31
External consultant physician	142 (41.3)	214 (49.4)	0.02

Values are represented as number (%), unless stated otherwise.

*SOFA* sequential organ failure assessment, *WhWd* withhold or withdraw treatment, *QOL* quality of life. By convention, a SOFA organ sub-score of 3 or more was considered as organ failure

Among the 344 Wh patients, 105 only had “do not start” instructions prohibiting cardiopulmonary resuscitation (91), renal replacement therapy (72), inotrope use (64), endotracheal (58) or non-invasive (20) ventilation, surgery (51), blood product transfusion (35), antibiotics (17), and oxygenation (1). Compared to the 239 Wh patients who had “do not start” and/or “do not increase” instructions, these 105 patients had lower median SAPS II (49 vs 57), total SOFA score (4 vs 6), rate of respiratory (22 vs 40 %) and circulatory (17 vs 35 %) failures, and death rate (33 vs 57 %) in the ICU (*p* < 0.01). Conversely, chronic respiratory diseases were more frequent in this group (48 vs 33 %).

### Patients’ outcome

Of the 4812 patients without WhWd measures, 4264 (89 %) left the ICU alive, while 548 (11 %) died in the ICU.

Of the 777 WhWd patients, 193 (25 %) survived and were discharged from the ICU, whereas 584 (75 %) died in the ICU. The median (IQR) time from withholding/withdrawal completion to death in the ICU was 2 days (1–6) after withholding (172/344 deaths after Wh, 50 %), and 1 day (0–3) after withdrawal (412/433 deaths after Wd, 95 %). Sixty more WhWd patients died on the ward after discharge from the ICU, which means that 133 patients (17 % of all WhWd patients) were discharged alive from the hospital (Fig. 1).

## Discussion

Contrary to ethicists [21, 22, 31], many intensivists clearly distinguish between withholding and withdrawal decisions, with the former being perceived as more “passive” [3, 4, 10, 32, 33]. Physicians’ unwillingness to withdraw life-sustaining therapy has been previously associated with religion, culture, experience, and gender [14, 33–37]. Rather than focusing on differences between centers, our study aimed to identify the conditions that specifically led to withdraw and/or withhold therapy. By establishing a three-level hierarchy of decisions (“stop” > “do not increase” > “do not start”), we demonstrated that more “active” limitations involved patients with acute organ failures, high severity indexes, and great dependence on life-sustaining therapy. Brain-injured patients were also more likely to undergo a withdrawal procedure, whereas patients with chronic respiratory disease, pre-existing disability affecting autonomy or cognition, and/or respiratory failure on admission had treatment preferentially withheld than withdrawn. Whatever the level of limitation applied, the patient’s wishes were unknown in more than three quarters of cases at the time of the decision-making. Thus, decisions to limit treatment were predominantly based on medical judgment. Amazingly, while withdrawals of life-sustaining treatment (with hastening of death as a possible risk) were thoroughly argued, a consultant physician was involved at the level of the decision-making in less than half cases.

There is an extensive literature based on questionnaire and epidemiological surveys exploring attitudes of intensive care physicians on forgoing treatments. Influenced by multiple factors, practices vary considerably between and within countries [3, 4, 10, 32, 38–40]. Although most Western physicians consider withholding/withdrawing treatment usual, respondents to a recent multinational survey in Asia reported that they commonly withheld (70.2 %) but rarely withdrew (20.7 %) treatments [10]. In a hypothetical scenario of post-anoxic coma with septic shock, Asian physicians are less likely to withhold or withdraw life-sustaining treatments, and more likely to “do everything” (53.8 %) than those in Western countries (USA < 40 %, Southern Europe < 30 %, Canada < 20 %, Australia < 10 %, Northern and Central Europe < 10 %) [10, 32, 38]. In 37 ICUs from 17 European countries (2000–2001), Sprung found that 76 % of deaths were preceded by some kind of limitation, with a clear downward North/South tendency between regions. Amazingly, the French centers who volunteered to take part in the survey could not obtain approval from their ethics committee [3]. However, despite inter-unit variability, our data are close to those collected in 1997 by the French LATAREA group in terms of proportion of ICU patients undergoing WhWd measures (14 vs 11 %) or dying after a decision to limit life-supporting therapies (52 vs 53 %) and proportion of withholding/withdrawal decisions (41/59 vs 44/56 %) [2]. These two French surveys were completed 16 years apart, the former (LATAREA) before and the latter (EPILAT) after enactment of the Leonetti law. Considering such apparently limited impact on WhWd rates (despite the higher median age and SAPS II in our study population), one could argue that the law provided a legal framework for practices that already existed informally.

Because most patients in the ICU lack decision-making capacity, WhWd discussions are often shared between physicians, nurses, and family members or relatives acting as surrogates and representing the patient’s values and preferences [21]. One important finding from this study is that decisions regarding WhWd are primarily founded on medical judgment. The low level of patients being directly or indirectly involved in the decision-making (23 %) may reflect that many were unable to express their preferences once hospitalized, and/or that they did not anticipate such conditions of being before admission. While French Parliament unanimously passed the Leonetti law in 2005 after a long and highly publicized debate, the prevalence of advance directives or designated trusted person remains low. The availability and legal value of advance directives widely differ by country and show the balance between the culture of patient autonomy and that of paternalism in medical care [41]. In a survey of US citizens aged 60 years or older who have died of any cause between 2000 and 2010, the proportion of decedents with advance directives increased from 47 % in 2000 to 72 % in 2010 [42]. According to the French law, the procedure should also involve an independent corroboration of the diagnosis and prognosis by an external physician who is missing in more than half the cases in our study. The rate of external corroboration is only slightly higher in case of treatment withdrawal (vs withholding). Whereas hospital specialists know best in their particular domains about the prognosis for diseases, it could be hypothesized that only skilled intensivists could assess the benefit–risk/burden balance of life-sustaining therapies such as ventilatory support, inotrope use, renal replacement therapy, or extracorporeal oxygenator. Because many hospitals only have one ICU, referring to an independent and relevant arbitration may be challenging.

Treatment limitations in brain-injured patients differ from those applied to patients with end-stage irreversible diseases. In the former category (post-anoxic coma, stroke, head trauma), patients are rarely or never conscious at the time of the decision-making and cannot be involved in the discussion. Moreover, continuation of treatment may prolong life for months or years at the cost of being in a severely disabled state that such patients would not have accepted [41]. In our study, brain-injured patients qualified for a WhWd procedure, who empirically had the poorest ability to participate directly in decision-making, were more likely to undergo withdrawal rather than withholding of treatment compared to patients with non-neurologic diseases. By comparison, patients with chronic respiratory diseases, pre-existing limited autonomy, and/or respiratory failure as reason for admission had treatment preferentially withheld than withdrawn in this study. One potential explanation is that prognostic indexes based on several factors in combination may predict outcome with better accuracy in neuro-critical care than in other areas in medicine [41, 43–46]. In case of brain injury, the predicted outcome measure is either death or poor functional fate. For patients with congestive cardiac failure, obstructive bronchitis, cirrhosis, kidney disease, or cancer, it is rarely possible to prognosticate with certainty that a chronically ill subject would not survive an acute episode [47]. These patients need to undergo a time-limited trial of intensive care prior to any prognostication or WhWd decision [13, 31, 48]. However, most prediction models were not developed with the specific aim of informing end-of-life decisions [12, 41].

Limiting treatments in critically ill patients does not mean forgoing chances of survival. In the ETHICUS study, the rate of patients discharged alive from the hospital after withholding and withdrawal was 11 % (of 1594) and 1 % (of 1398), respectively [3]. In the French LATAREA study, 43 % of the Wh patients and 8 % of the Wd patients left the ICU alive [2]. A recent study from Norway reported a survival rate on hospital discharge of 37 and 0 % after withholding and withdrawal, respectively [49]. Our own survival rates on hospital discharge were 37 % after withholding and 2 % after withdrawal. Limitations did not solely involve patients who might die according to the physicians’ judgment. Even though withholding and withdrawing therapy have been considered ethically equivalent [21, 22, 31], our survey showed that in reality the more “active” limitations were associated with sudden and severe pathologies and the more “passive” with chronic diseases affecting respiration, autonomy, or cognition. Rather than ratifying a foretold death, the intention of the withholding decision was in some circumstances to let nature take its course toward death or life while avoiding non-beneficial and burdening therapies.

The current study has several strengths. Prospectively carried out in a large number of units throughout a single country, it identified conditions specifically associated with withholding or withdrawal of treatment. Limitations of treatment were described in details separating “do not start,” “do not increase,” and “stop” instructions. The study also showed that the rights to dispose of one’s health conferred on citizens by law (advance directives, trusted person) were under-used, and as a result, that decisions remained under physicians’ authority.

It also has limitations. First, it is uncertain whether the units involved in the study were representative of French practices. Second, information about sedation and analgesia given during the WhWd procedure (particularly Wd) was not collected. Third, neither the temporal steps of the WhWd procedure (first discussion, consensus reached within the staff, agreement obtained from families and/or relatives, implementation of the measures agreed) nor the prognostic indexes used to select patients for limitations were recorded.

## Conclusions

In our study involving 43 French ICUs, more than half deaths occurred after a formal decision to withhold or withdraw therapies deemed non-beneficial. Brain-injured patients were more likely to undergo a withdrawal procedure, whereas patients with chronic respiratory disease and pre-existing disability affecting autonomy or cognition had treatment preferentially withheld than withdrawn. While the law authorizing such practices was passed in 2005, the prevalence of advance directives and designated trusted persons remains low. An external consultant was involved in less than half of all decisions. Because patients’ wishes are rarely known at the time of the decision-making, limitations remained primarily based on medical judgment.

## Abbreviations

BDD: brain determination of death
CDD: circulatory determination of death
CI: confidence interval
FiO2: fraction of inspired oxygen
ICU: intensive care unit
IQR: interquartile
MD: missing data
OR: odds ratio
SAPS: Simplified Acute Physiology Score
SD: standard deviation
SOFA: Sequential Organ Failure Assessment
WhWd: withholding or withdrawing treatments
